# Gluten and anxiety: a difficult balance in people with celiac disease

**DOI:** 10.1192/j.eurpsy.2023.1436

**Published:** 2023-07-19

**Authors:** M. A. Robea, A. Ciobica, C. Stanciu, A. Trifan

**Affiliations:** 1 Institute of Gastroenterology and Hepatology, “St. Spiridon” Emergency Hospital; 2 Biology, Faculty of Biology, “Alexandru Ioan Cuza” University of Iasi; 3 Romanian Academy, Center of Biomedical Research; 4 Gastroenterology, University of Medicine and Pharmacy “Grigore T. Popa”, Iasi, Romania

## Abstract

**Introduction:**

Celiac disease (CD), triggered by gluten ingestion, occurs in people genetically predisposed to develop this chronic autoimmune condition. The triggering environmental factor, gluten, is known as a protein present in wheat, rye and barley. In recent decades, specialists have found more knowledge about the disease mechanisms, how it develops and other disturbances which accompany it. The CD was considered a pediatric main gastrointestinal disorder, associated with symptoms of abdominal pain, diarrhea, constipation and bloating, and characterized by damage to the villi of the small intestine. People with CD may experience anemia, fatigue, osteopenia or osteoporosis, bone fracture, neurological and psychiatric problems beside anxiety as depression, ataxia, neuropathy. However, the results of several studies conducted on the fact that people with CD have an increased level of anxiety are mixed.

**Objectives:**

The present work is highlighting the importance of observing the anxiety levels in people diagnosed with CD beside the suitable interventions in reducing it.

**Methods:**

For our study scientific databases were screened using certain keywords and combinations of it as: ”celiac disease”, ”gluten”, ”gastrointestinal disorder”, ”treatment”, ”neurological problems”, ”anxiety”. Inclusion criteria were studies that (1) investigated anxiety levels in CD people, (2) reported gender results, (3) were written in English, and (4) were published within the last 20 years.

**Results:**

In some cases, as main intervention in CD, gluten removal from the people’s diet usually reported improvements of the present symptoms. In addition, data from literature are describing a higher level of anxiety in females compared to males diagnosed with CD. This can be a consequence of females concerns about how they can manage the CD issues and, especially, what this is bringing in their lifestyle. On the opposite, there are reports which showed that demographic parameters (gender, age, education) are not associated with CD presence.

**Conclusions:**

The balance between CD and anxiety needs to be more investigated in order to identify and fully understand what is the background mechanism and how this can be regulated through specific interventions.

**Disclosure of Interest:**

M. Robea Grant / Research support from: ,Platformă multidisciplinară de cercetare-dezvoltare medicală în regiunea N-E,,, acronim: CENEMED, cod mySMIS: 127606, proiect cofinanțat prin Programul Operațional Competitivitate, Axa Prioritară: Cercetare, Dezvoltare tehnologică și Inovare (CDI)., A. Ciobica: None Declared, C. Stanciu: None Declared, A. Trifan: None Declared

